# Time- but not sleep-dependent consolidation promotes the emergence of cross-modal conceptual representations

**DOI:** 10.1016/j.neuropsychologia.2014.08.021

**Published:** 2014-10

**Authors:** Nora Hennies, Penelope A. Lewis, Simon J. Durrant, James N. Cousins, Matthew A. Lambon Ralph

**Affiliations:** a Neuroscience and Aphasia Research Unit, School of Psychological Sciences, University of Manchester, Zochonis Building, Brunswick Street, Manchester M13 9PL, UK; b School of Psychology, University of Lincoln, Lincoln LN6 0BG, UK

**Keywords:** Memory consolidation, Sleep, Category learning, Abstraction, Cross-modal object representations

## Abstract

Conceptual knowledge about objects comprises a diverse set of multi-modal and generalisable information, which allows us to bring meaning to the stimuli in our environment. The formation of conceptual representations requires two key computational challenges: integrating information from different sensory modalities and abstracting statistical regularities across exemplars. Although these processes are thought to be facilitated by offline memory consolidation, investigations into how cross-modal concepts evolve offline, over time, rather than with continuous category exposure are still missing. Here, we aimed to mimic the formation of new conceptual representations by reducing this process to its two key computational challenges and exploring its evolution over an offline retention period. Participants learned to distinguish between members of two abstract categories based on a simple one-dimensional visual rule. Underlying the task was a more complex hidden indicator of category structure, which required the integration of information across two sensory modalities. In two experiments we investigated the impact of time- and sleep-dependent consolidation on category learning. Our results show that offline memory consolidation facilitated cross-modal category learning. Surprisingly, consolidation across wake, but not across sleep showed this beneficial effect. By demonstrating the importance of offline consolidation the current study provided further insights into the processes that underlie the formation of conceptual representations.

## Highlights

• We examine how conceptual representations evolve offline.
• We use an abstract, cross-modal category learning task to mimic concept formation.
• 24h consolidation and 12h consolidation across wake facilitate cross-modal category learning.
• 12h consolidation across sleep has no beneficial effect.

## 1 Introduction

Every day we automatically discriminate between hundreds of objects, and assign meaning to them. This process often requires the integration of information from different modalities. For instance, when discriminating a donkey from a mule, information about its shape, the colour of its fur, or its location overlaps between the two species and is therefore, individually, not sufficient for correct categorisation. However, when the information is integrated, the two exemplars can be pulled apart, which allows us to rapidly discriminate between them. To account for this ability and to allow for generalisation to novel objects or situations, the conceptual representation of an object enables the capture of regularities and variations across the different modalities (Medin & Rips 2005; Lambon Ralph, Sage, Jones, & Mayberry 2010; Lambon Ralph, 2014). The formation of many real-world concepts therefore seems to depend on two crucial mechanisms: the abstraction of the statistical variation across exemplars and the integration of information from different modalities (Rogers & McClelland, 2004; Lambon Ralph et al., 2010). How new conceptual representations form with on-going category training has been studied in great detail (Ashby & Maddox 2005; Smith & Minda, 2002; Kumaran, Summerfield, Hassabis, & Maguire 2009; Jiang et al., 2007; van der Linden, Murre, & van Turennout, 2008; van der Linden, van Turennout, & Indefrey, 2010; van der Linden, van Turennout, & Fernandez, 2011). To date, however, very little research has focused on how conceptual representations evolve over time (Djonlagic et al., 2009).

Memory consolidation describes a post-encoding process of reorganisation, through which new memories become stabilised and integrated into long-term memory (Frankland & Bontempi, 2005). In addition to its stabilising effect, memory consolidation, including that which occurs across sleep, has been associated with a qualitative change of memories towards more abstract and general representations (McClelland, McNaughton, & O’Reilly, 1995; Winocur, Moscovitch, & Bontempi, 2010; Walker & Stickgold, 2010). Specifically, memory consolidation has been shown to facilitate the integration of distinct elements into coherent constructs (Walker & Stickgold 2010; Kuriyama, Stickgold, & Walker, 2004; Ellenbogen, Hu, Payne, Titone, & Walker, 2007; Lau, Alger, & Fishbein, 2011). This has been demonstrated, for example, using a relational memory task in which participants were taught objects pairs, embedded in a hidden hierarchy (Ellenbogen et al., 2007). Consolidation across sleep promoted the links between individual items and the hierarchical structure. A similar but weaker benefit was observed during sleep-independent consolidation. Other evidence suggests that sleep-dependent consolidation facilitates the incorporation of newly learned information into the network of pre-existing knowledge (Tamminen, Payne, Stickgold, Wamsley, & Gaskell, 2010). Besides this integrative function, memory consolidation also seems to play a role in the abstraction of rules (Gomez, Bootzin, & Nadel, 2006; Wagner, Gais, Haider, Verleger, & Born, 2004; Sweegers, Takashima, Fernandez, & Talamini, 2013), statistical patterns (Fischer, Drosopoulos, Tsen, & Born 2006) and the generalisation of information (Tamminen et al., 2010). Durrant, Taylor, Cairney, and Lewis et al. (2011); and Durrant, Cairney, and Lewis, (2012), for instance, showed that sleep-dependent and, less strongly, sleep-independent consolidation promoted the abstraction of an implicit probabilistic structure in sequential auditory stimuli. Given that both the integration of information as well as the abstraction of statistical patterns seem to present fundamental mechanisms in the formation of conceptual representations, a key target of the current study was to explore how memory consolidation, possibly dependent on sleep, facilitates these aspects of concept formation.

A study by Maddox et al. (2009) partially tackled this question by investigating the effect of sleep deprivation on information-integration category learning. The information-integration category learning task has been extensively studied in category learning (Ashby & Maddox, 2005). Key features of this task are that: the category structure cannot be easily verbalised; categorisation accuracy is only maximised when information from two or more stimulus dimensions is integrated at some predecisional stage (Ashby & Gott, 1988); and, categories can display strong within-category variation along different dimensions. The information-integration category learning task, therefore, nicely mimics the basic mechanisms involved in the formation of many natural concepts, as described above. The study conducted by Maddox et al. showed that sleep deprivation led to an overall performance deficit in the information integration category learning task. In the context of the current study more importantly, they also found a significant performance increase in the information-integration category learning task over a 24-hour off-line consolidation period, in which participants received a normal night of sleep. This performance increase cannot be directly attributed to a consolidation benefit as there was no control group, which performed the task without a consolidation break, but it does suggest that sleep may benefit this type of category-learning.

The current study investigated the effect of consolidation on the emergence of cross-modal category representations in more detail. The formation of conceptual representations in real life is usually unintentional - fostered by the incidental exposure to category members. This type of category learning is assumed to be mediated by an implicit, procedural system (Ashby & Maddox 2005; Smith et al., 2012). By modifying the information-integration category learning task (Ashby & Gott 1988), we attempted to mimic the emergence of natural concepts, reduced to its two key mechanisms: the integration of cross-modal information and the abstraction of statistical regularities. We developed a paradigm in which an information-integration structure, across two different sensory modalities (auditory and spatial), was learned through a simple rule-based categorisation task. The abstract nature of this task prevented participants from drawing on prior knowledge and allowed us to track category learning from its very early stages. We conducted two experiments to investigate the effect of time-dependent consolidation (Experiment A) and the effect of sleep-dependent consolidation (Experiment B) on category learning. We predicted that the underlying information-integration category structure would be picked up implicitly during the training and enhanced by time- and sleep-dependent consolidation.

## 2 Methods

### 2.1 Participants

Experiments A and B involved 26 participants each. Informed consent was obtained from all participants prior to the study, approved by the University of Manchester Research Ethics Committee. Participants were not familiar with Asian orthographic characters, had normal or corrected-to-normal vision and hearing, and no prior history of psychiatric, learning or sleep disorders. Participants were required to be free of psychological drugs, alcohol and caffeine, and to refrain from daytime napping for 24 h preceding and throughout the study period. In Experiment A participants were randomly assigned to either a 15 min consolidation group (15 min group, *n*=13, mean age: 24.00, S.D.±4.51, 6 F) or a 24 h consolidation group (24 h group, *n*=13, mean age: 24.14, S.D.±3.59, 5 F). In Experiment B participants were randomly assigned to either a 12 h day consolidation group (12 h day, *n*=13, mean age: 19.50, S.D.±1.22, 11 F) or a 12 h night consolidation group (12 h night, *n*=13, mean age: 20.00, S.D.±1.30, 11 F).

### 2.2 Stimuli and stimulus generation

Each stimulus was defined by a combination of spatial, auditory and visual information, and belonged to one of two categories. In the spatial dimension, each stimulus was characterised by a specific location along the horizontal screen axis, in the auditory dimension by a particular pitch (between 200 and 1000 Hz) and in the visual dimension by an image of an Asian orthographic character. Category assignment was predefined based on obvious image characteristics. However, the same category assignment could be achieved by integrating the information about location and pitch.

Stimuli were first created in spatial and auditory dimensions, in which the category structure was based in terms of an information-integration structure (Ashby & Gott 1988). The visual dimension was added to each stimulus in a second step. Stimuli were generated by drawing 72 random samples from each of two bivariate normal distributions, forming the two stimulus categories. Distribution parameters are shown in Table 1. Transformations of the original values (*x*,*y*) into the two stimulus dimensions, space (*x*′, min: −400 pixels, max: 400 pixels from the centre) and tone (*y*′, min: 200 log_2_Hz, max: 1000 log_2_HZ), were performed according to Formulas (1) and (2).

(1) $$x\prime =\;\frac{\left(x+400\right)(x_{max}^{\prime }-\;x\prime_{min})}{800}+x_{min}^{\prime }$$

(2) $$y\prime =\;y\prime_{\min}\times 2^{(y-200)/(800)\times log_{2}\left(y_{\max}^{\prime }/y_{\min}^{\prime }\right)}$$

**Fig. 1:**
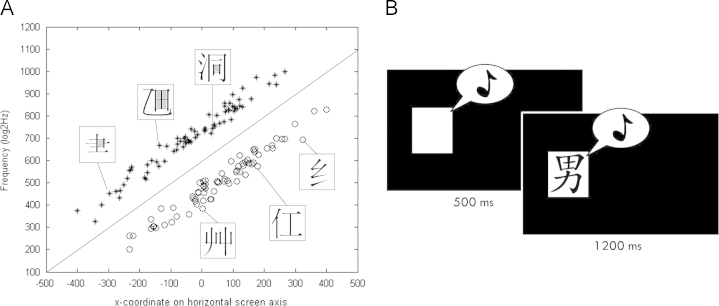
Visualisation of the category structure (A) and the CMCL-task trial structure (B). (A) An asterisk denotes stimuli from category 1. Stimuli from category 2 are indicated by open circles. The abscissa corresponds to the location along the horizontal screen axis, the space dimension of a stimulus. The ordinate corresponds to the pitch (frequency in log2(Hz)), the auditory dimension of a stimulus. In this two-dimensional space the category structure is an information-integration structure (Ashby & Gott 1988). Each two-dimensional stimulus is paired with an image of an orthographic character. Stimuli of category 1 are paired with characters, which have an enclosed space (for visualisation purpose coloured in grey); stimuli of category 2 are paired with open shaped characters. Category membership can be detected either based on a simple rule regarding the image (‘open’, ‘closed’) or by integrating information on location and tone. (B) Every trial started with the simultaneous presentation of just the auditory and the spatial dimension of a stimulus for 500 ms, before the orthographic character appeared. The three-dimensional stimulus was presented for 1200 ms.

To visualise the category structure, each stimulus can be represented graphically by a point in the two-dimensional stimulus space as shown in Fig. 1. In a second step, each two-dimensional stimulus was paired with an image of an Asian orthographic character. All orthographic characters were distinct and could be easily distinguished. Stimuli of category 1 were paired with characters, which had at least one enclosed space; stimuli of category 2 were paired with characters without an enclosed space (see Fig. 1 for examples). Important to note is that this category structure allowed correct categorisation of a stimulus based on its image (‘open’ or ‘closed’) as well as based on integrating its information coded on location and tone dimensions. Stimuli for the CMCL-task and the explicit memory tasks were randomly drawn from this pool of stimuli, such that 50% fell in each category.

### 2.3 Experimental tasks

All tasks were presented using Cogent 2000 developed by the Cogent 2000 team at the FIL and the ICN. They were written and executed using MATLAB^©^ 7.5 on a desktop PC with dual core Xeon processor. Sounds were heard through a pair of Sennheiser^©^ HD207 noise-cancelling headphones. Stimuli were presented on a 17″ computer screen on black background.

#### 2.3.1 Cross-modal category learning task

For each participant 48 stimuli (24 stimuli per category) were randomly drawn from the pool of stimuli. Each time it was confirmed by plotting the selected stimuli that the selected set was representative of the distribution. The cross-modal category learning (CMCL) task, in both experiments, consisted of four or five repeated blocks depending on the group. In each block all 48 stimuli were presented once, in randomised order. Every trial started with the simultaneous presentation of the auditory and spatial information of the stimulus. The spatial information was indicated by a white square (4×4 cm^2^) appearing at the specific location. After 500 ms the orthographic character was presented within the white square. The 3-dimensional stimulus was presented for 1200 ms. Using correspondingly labelled keys on the computer keyboard (C: closed, O: open), participants were instructed to categorise the stimulus as quickly and accurately as possible according to the image into one of the two categories ‘open’ or ‘closed’. The last response given during the 1700 ms stimulus presentation period was counted and the reaction time for this response was recorded. This gave participants the chance to correct their responses and therefore encouraged participants to respond more quickly. Stimulus presentation was followed by 800 ms blank screen and initiation of the next trial. After each block participants had a self-determined break and received feedback on their average reaction time, the number of mistakes and if they had improved compared to previous blocks. Participants were not aware of the underlying implicit category structure and the aim of the study.

Crucial to our design was that the integrated information about the location and the tone of a stimulus, which always preceded the image, actually predicted the category membership of the image. Hence, use of the spatial and auditory information for categorising the stimuli would be reflected in accelerated response times. We hypothesised that, with training, participants would start to abstract the underlying cross-modal category structure and use this information for categorisation. A relative decrease in the average response time compared to the control (see next paragraph) served as indicator for the emergence of category knowledge. While the first few blocks of the CMCL-task were considered as training, the final block served as test block and was used for the analysis.

#### 2.3.2 Control task

The control task was used to determine individual reaction time baselines for categorising the images of the CMCL stimuli into the two categories ‘open’ or ‘closed’. In the control task the same 48 stimuli that were used in the CMCL-task were presented once in their visual dimension only (i.e., the orthographic character without location or tone information). Each trial started with the presentation of a white square (4×4 cm^2^) in the middle of the screen. After 500 ms the orthographic character was displayed for 1200 ms within the square. No spatial or auditory information was given. Stimulus presentation was followed by 800 ms blank screen and initiation of the next trial. Task instructions and measures were identical to the CMCL-task. As control trials differed from CMCL-task trials only in the absence of auditory and spatial information, response time differences between the control and the CMCL-task could be attributed to the use of this information for categorising the stimuli. The difference in the average response time between the control and the last block of the CMCL-task served as measure for category learning in both experiments.

#### 2.3.3 Explicit memory tasks

Three additional tasks were conducted to investigate whether explicit memory components contributed to the reaction time performance in the CMCL-task. In all tasks responses were given by pressing correspondingly labelled keys on the keyboard. No feedback was received. Novel stimuli were randomly drawn from the pool of the remaining stimuli, whose generation was described in Section 2.2 with the constraint that half fell in each category.

1. Recognition task: This task addressed how well individual stimuli were remembered. Therefore, 24 out of the 48 CMCL-task stimuli were randomly chosen with the constraint that half fell in each category and presented simultaneously in all 3 dimensions intermixed with 24 novel stimuli. Participants were instructed to indicate if they recognised each stimulus. Each trial was response terminated.
2. Association task: In this task, the memory for the combination of the visual dimension (image) with the auditory and spatial information was tested. Participants were tested on the 48 CMCL-stimuli. 24 of these stimuli were presented in their original three-dimensional combination. The other 24 stimuli were recombined. Therefore we kept the combination of spatial and auditory information fixed and shuffled the images between these stimuli within one category. Of the 24 stimuli presented as familiar items in the recognition task, 12 were recombined and 12 were presented in their original combination. We did not control for the similarity distance between the old and the recombined items. The stimuli were presented, one at a time, in randomised order. Participants were instructed to indicate for each stimulus if the image and the space-tone combination was the same as in the CMCL-task or not. Each trial was response terminated.
3. Categorisation task: This task was used to test if participants could correctly categorise stimuli based on their spatial and auditory information only. The CMCL-task stimuli were presented once, in random order, under this categorisation condition. In each trial location and tone information of a stimulus was simultaneously presented for 1700 ms and participants were instructed to indicate if the stimulus would have an ‘open’ or a ‘closed’ shape in its visual dimension.

### 2.4 Procedure

**Fig. 2:**
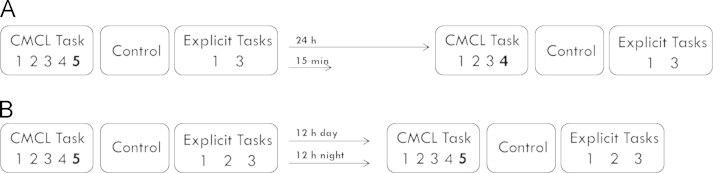
Schematic illustration of the experimental procedures of experiments A and B. Both experiments consisted of two experimental sessions, separated by a consolidation interval. Consolidation interval characteristics differed between conditions as indicated above the arrows. Each session comprised several blocks of the cross-modal category learning (CMCL) task, with the respective final block serving as test block of interest, the control task and two (in Experiment B three) additional memory tasks.

Both experiments consisted of two experimental sessions, which were separated by a consolidation interval. This interval differed across four conditions. In Experiment A, the two experimental sessions were either separated by 24 h (±30 min) or by 15 min. Both sessions took place between 10 a.m. and 5 p.m. (mean starting time: 13:30, S.D.±2 h 14 min). In Experiment B, the two sessions were separated by a 12 h (±1 h) interval which took place either during the night or during the day. Sessions took place in the morning at 8 a.m. (±1 h) and in the evening at 8 p.m .(±1 h). The 12 h day group completed the first session in the morning and the 12 h night group completed the first session in the evening, followed by a normal night of sleep. A schematic illustration of the procedure is shown in Fig. 2.

Session one started with a brief training round familiarising participants with stimuli and instructions. Subsequently participants performed the CMCL-task. Stimuli for the CMCL-task were randomly selected for each participant from the pool of 72 stimuli in each category. The CMCL-task was followed by the control task and the explicit memory tasks. The association task was performed by participants in Experiment B only. After completion of this session, which took about 45 min, participants were instructed to leave and carry on with their usual daily activities (12 h wake group, 24 h group), to have a 15 min break outside of the testing room (15 min group) or to return home and have a normal night of sleep (12 h night group). Session two started with the CMCL-task on 48 novel stimuli. In experiment A the CMCL-task in this session included 4 blocks only. Subsequently participants performed the control and the explicit memory tasks. This session lasted for 45 min. At the beginning of both sessions participants of Experiment B filled out a Karolinska Sleepiness Scale (KSS).

### 2.5 Statistical analysis

The same statistical analysis was conducted on Experiments A and B. Category learning was assessed in each session by comparing response times of the last CMCL-block with the corresponding control. Therefore, a 2×2 mixed analysis of variance (ANOVA) with within-subjects factor task (CMCL, control) and between-subjects factor group (Experiment A: 24 h and 15 min; Experiment B: 12 h day and 12 h night) was conducted, separately for each session, on the response times (in ms) to assess category learning before consolidation (session 1) and after consolidation (session 2). Simple effects were analysed using two-tailed paired t-tests. Since participants were instructed to categorise each image based on a simple visual rule, accuracy was expected to be at ceiling and not of interest for our study. Performance on the recognition task and the association task was assessed by calculating the sensitivity index (d′) as d′=z-score(hits) – z-score(false alarms) from the number of hits and the number of false alarms. In cases where maximum hits or no false alarms occurred, the common practice of reducing or increasing the proportion correct by the equivalent of half a trial was followed (Durrant, et al., 2012). 2×2 mixed ANOVAs with within-subjects factor session (session 1, session 2) and between-subjects factor group (Experiment A: 24 h and 15 min; Experiment B: 12 h day and 12 h night) were conducted on the sensitivity index, separately for each task. Performance on the categorisation task was assessed by calculating the number of trials on which the correct category membership was identified. A 2×2 mixed ANOVA with within-subjects factor session (session 1, session 2) and between-subjects factor group (Experiment A: 24 h and 15 min; Experiment B: 12 h day and 12 h night) was conducted on the number of correct trials. Data were analysed in SPSS 20. In all our results we considered *p*<0.05 as significant. All tests were two-tailed and Bonferroni corrected, unless stated otherwise.

## 3 Results

### 3.1 Experiment A

#### 3.1.1 CMCL-task

**Fig. 3:**
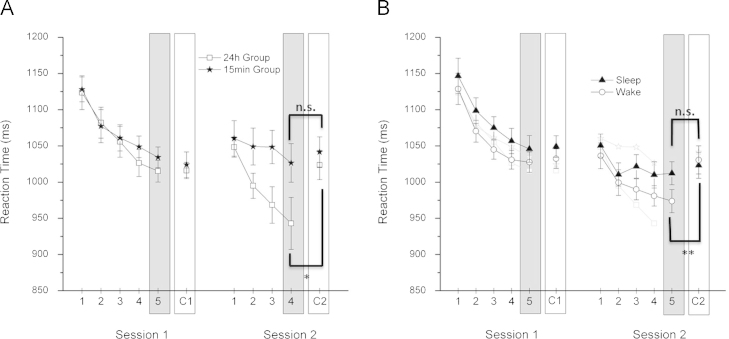
Reaction time results for Experiment A (24 h and 15 min group) and Experiment B (12 h day and 12 h night group). Average response times are shown for all blocks of the CMCL-task and the control (C), for each experimental session. Standard error bars are included. In each session the final block of the CMCL-task was considered as test block (grey box) and response time differences between this block and the control (white box) served as measure for category learning. (A) Session 2: The 24 h group performed significantly faster in the final CMCL-task block than in the corresponding control, indicating the use of integrated auditory and spatial information. This difference was not significant for the 15 min group. (B) Session 2: The 12 h day group showed a significant reaction time decrease in the CMCL-task block compared to the corresponding control. This difference was not significant for the 12 h night group. The data points plotted in light grey correspond to the response times of experiment A. *p<0.05, **p<0.01.

In Experiment A we sought to assess how a post-learning consolidation interval would influence cross-modal category learning. One participant of the 15 min group was excluded from the analysis as the response times deviated by more than three standard deviations from the group average. Results are shown in Fig. 3 A. In session one there was no difference in the response times of the CMCL-task and the control, *F*(1,23)=0.43, *p*=0.53, and no difference in the performance between the two groups, *F*(1,23)=0.48, *p*=0.50. The interaction between the factors task and group was also not significant, *F*(1,23)=0.57, *p*=0.46. These results suggested that before the consolidation interval, no category learning had taken place in either group. In session two there was again no main effect of group, *F*(1,24)=0.47, *p*=0.5. Importantly, however, in this session the overall response times for the CMCL-task were significantly lower (*M*=983.1 ms, SD=119.9 ms) than for the control (*M*=1032.3 ms, SD=73.0 ms), F(1,23)=7.22, *p*=0.01, indicating that information of the underlying information-integration category structure was used for categorisation. The interaction between task and group did not reach significance but displayed a trend, *F*(1,23)=3.38, *p*=0.08. Planned comparisons, using Bonferroni adjusted alpha levels of 0.025, showed that while for the 24 h group there was a significant difference in response times between the CMCL-task and the control, *t*(12)=2.82, *p*=0.015, this difference was not significant for the 15 min group, *t*(11)=0.74, *p*=0.47. Showing subtle but important differences between the two groups, these results suggest that category learning was dependent on the presence of the consolidation interval.

#### 3.1.2 Explicit memory tasks

Two explicit memory tasks were conducted at the end of each experimental session to investigate how well participants could remember the individual items (recognition task) and if they had acquired explicit knowledge about the underlying information-integration category structure (categorisation task). The results are summarised in Table 2. Performance did not differ between groups (Recognition task: *F*(1,23)=2.55, *p*=0.12; Categorisation task: *F*(1,23)=0.11, *p*=0.74), or sessions (Recognition task: *F*(1,23)=2.54, *p*=0.13; Categorisation task: *F*(1,23)=2.58, *p*=0.12) in either task, and the interaction between session and group was not significant (Recognition task: *F*(1,23)=0.61, *p*=0.44; Categorisation task: *F*(1,23)=1.92, *p*=0.18). These results suggest that the overall reaction time decrease in the CMCL-task compared to the control observed in the second session was not due to improved item recognition or improved explicit categorisation performance within the second session. Performance was above chance in the recognition task, using Bonferroni adjusted alpha levels of.0125, for each group in each session (*t*≥5.78, *p*≤0.001). Interestingly, for the categorisation task only the performance of the 24 h group in the second session was above chance (*M*=29.3, SD=6.2), using Bonferroni adjusted alpha levels of.0125, *t*(12)=3.08, *p*=0.01. All other performances were at chance level (*t*≤1.686, *p*≥0.118).

### 3.2 Experiment B

#### 3.2.1 CMCL-task

Experiment B sought to assess how post-learning sleep or wakefulness would influence cross-modal category learning. Results are shown in Fig. 3 B. The analysis of session one showed no significant difference between the response times of the CMCL-task and the control, *F*(1,24)=0.40, *p*=0.533, no difference between groups, *F*(1,24)=0.71, *p*=0.41, and no interaction between the factors task and group, *F*(1,24)=0.01, *p*=0.91. Consistent with the results of Experiment A, no abstraction of the underlying category structure seemed to have occurred before the consolidation interval. In session two we found no difference between groups, *F*(1,24)=0.47, *p*=0.5, but a significant main effect of task, *F*(1,24)=13.02, *p*=0.001. Response times were quicker for the CMCL-task (*M*=992.99 ms, SD=59.66 ms) than for the control (*M*=1026.95 ms, SD=66.19 ms). Importantly, there was also a significant interaction between task and group, *F*(1,24)=5.97, *p*=0.02. Simple effects analysis, using Bonferroni adjusted alpha levels of 0.025, revealed that this interaction was driven by a significantly lower response time for the CMCL-task (*M*=973.7 ms, SD=67.6 ms) than for the control (*M*=1030.7 ms, SD=69.9 ms) in the 12 h day group, *t*(12)=4.00, *p*=0.002. For the 12 h night group the difference between CMCL and control tasks was not significant, *t*(12)=0.89, *p*=0.39. In line with the results of Experiment A, these results suggest that category learning occurred in the second session and was modulated by the consolidation interval. Surprisingly, the 12 h consolidation interval including wake and not sleep seemed to have a beneficial effect on category learning.

#### 3.2.2 Explicit memory tasks

In additional to the recognition and the categorisation tasks used in Experiment A, Experiment B also tested how well participants could remember the association between the visual orthographic character and the correct space-tone information (association task). The results are summarised in Table 2. There was no difference between groups (Recognition task: *F*(1,24)=1.78, *p*=0.20; Association task: *F*(1,24)=0.14, *p*=0.72; Categorisation task: *F*(1,24)=1.85, *p*=0.19), or sessions (Recognition task: *F*(1,24)=0.02, *p*=0.89; Association task: *F*(1,24)=3.10, *p*=0.09; Categorisation task: *F*(1,24)=1.53, *p*=0.23), and no interaction between session and group in any task (Recognition task: *F*(1,24)=0.12, *p*=0.73; Association task: *F*(1,24)=0.45, *p*=0.51; Categorisation task: *F*(1,24)=0.09, *p*=0.77). In the recognition task performance was above chance, using Bonferroni adjusted alpha levels of.0125, for both groups in both sessions (*t*≥5.78, *p*≤0.001). In the association task, only performance of the 12 h night group in the second session was above chance, *t*(12)=3.311, *p*=0.006, all other scores were at chance (*t*≤2.842, *p*≥0.015). In line with the finding of Experiment A, only the performance of the 12 h day group on the categorisation task in the second session was above chance, *t*(12)=3.5, *p* =0.004. All other scores were at chance (*t*≤2.115, *p*≥0.06). Interestingly, in both experiments, only the group, which showed a significant effect in the CMCL-task, performed above chance in the categorisation task. This finding suggests that, as expected, our reaction time measure captured the initial steps in the emergence of category knowledge.

#### 3.2.3 Circadian effects

As previous research has demonstrated that circadian rhythms may interact with memory formation, there is a potential danger of circadian confounds in memory studies (Siegel, 2001, Gerstner and Yin, 2010). Since experimental sessions were conducted at different times of day (e.g., in the morning after sleep and in the evening after wake), it is possible that our results were influenced by circadian factors. To test for this possibility, we conducted a 2×2 mixed ANOVA with factors time of day (evening, morning) and group (12 h day, 12 h night) on the response times of the control task, as performance on this task was expected to be constant between groups and sessions. Importantly, there was no main effect of time of day, *F*(1, 24)=1.691, *p*=0.206. We further found no main effect of group, *F*(1,24)=0.046, *p*=0.832, and no interaction between time of day and group, *F*(1,24)=2.240, *p*=0.148. Circadian influences were assessed using the same ANOVA on alertness measures of the KSS. Results showed again no main effect of the time of day, *F*(1,24)=0.008, *p*=0.930, no difference between groups, *F*(1,24)=0.004, *p*=0.951 and no interaction, *F*(1,24)=1.786, *p*=0.194. These results suggest that the differences in performance observed after retention across intervals including wakefulness or sleep were not due to circadian variations.

## 4 Discussion

The current study investigated the influence of time- and sleep-dependent consolidation on the acquisition of cross-modal conceptual representations. Participants learned to distinguish between members of two abstract categories based on a simple one-dimensional rule (in form of an orthographic character). Underlying the task was a hidden, more complex indicator of category structure, which required the integration of information across two sensory modalities. The response times in both experiments demonstrated that, at the end of the second session, participants benefitted from this additional cross-modal information, indicating that at least some initial category learning had occurred. The results of Experiment A suggested that this learning was dependent on the presence of a consolidation interval. In Experiment B we found that the state of consciousness during this consolidation interval had an impact on the consolidation that occurred. The results of the additional explicit memory tasks did not reveal any performance differences between groups or sessions – indicating that the CMCL reaction time-based task is more sensitive to the initial phases of category learning. Overall, the current data suggest that our task captured the very early stages in the emergence of cross-modal categorical representations.

Our results of Experiment A are in line with the performance increase observed by Maddox et al. (2009) in an information-integration category learning task over a 24 h consolidation period . The current study extends this finding by providing evidence that this type of category learning is indeed facilitated by post-exposure consolidation. The Information-integration category learning task is assumed to be mediated by a system that proceeds relatively automatically and without explicit awareness (Ashby & Maddox, 2005; Helie, Waldschmidt, & Ashby, 2010). There is strong evidence in the literature that consolidation benefits skill learning. This has mainly been demonstrated for motor memory (Brashers-Krug, Shadmehr, & Bizzi, 1996; Albouy et al., 2008; Dayan & Cohen 2011), sequencing (Karni, Tanne, Rubenstein, Askenasy, & Sagi, 1994; Press, Casement, Pascual-Leone, & Robertson, 2005; Robertson, Press, & Pascual-Leone, 2005) and visuo-motor (Krakauer, Ghez, & Ghilardi, 2005; Reis et al., 2013) tasks. In addition to the abstraction of statistical patterns or rules, which often presents a crucial component to skill learning, the information-integration category learning task requires the integration of information at a predecisional stage. Perhaps for the first time, the current study demonstrated that cross-modal probabilistic category learning also requires post-exposure consolidation across time. During consolidation, memory representations are assumed to be restructured (McClelland et al., 1995; Frankland & Bontempi, 2005), and this process has been associated with a qualitative change in which information is unitised and general patterns emerge (McClelland et al., 1995; Walker & Stickgold, 2010). Because the consolidation benefit only emerged after additional training, reorganisation of memory representations during the consolidation interval may have led to subtle changes, which then allowed a more effective abstraction of the underlying category structure during subsequent training.

A beneficial effect of sleep on simple procedural memory tasks, such as perceptual or motor learning (Gais, Plihal, Wagner, & Born, 2000; Walker, Brakefield, Morgan, Hobson, & Stickgold, 2002; Walker et al., 2003; Huber, Ghilardi, Massimini, & Tononi, 2004) is well established. Much less is understood about the role of sleep in more complex skill learning tasks, which involve the abstraction of rules or patterns, and the evidence up to date is inconclusive. While some studies show a beneficial effect of sleep-dependent consolidation (Fischer et al., 2006; Durrant et al., 2011; Durrant et al., 2012; Debas et al., 2010), other studies demonstrated consolidation benefits that are independent of sleep (Robertson, Pascual-Leone, & Press 2004; Nemeth et al. 2010) or even specific to wakefulness (Song, Howard, & Howard, 2007). The impact of sleep on probabilistic category learning was investigated by Djonlagic et al. using the weather prediction task (2009). This study showed a sleep-dependent benefit on category learning, which seemed to be mediated by rapid eye movement (REM) sleep. In the current study we found a clear dissociation between day-time and sleep-related processes but in contrast to Djonlagic et al., only consolidation across wake facilitated category learning. Taken together, the results from Experiments A and B suggest that a certain amount of wake was necessary in order for the consolidation benefit to occur in our specific task.

Similar to our results, a recent study conducted by Werchan and Gomez (2013) in young children showed a beneficial effect of wake but not sleep on the generalisation of word learning. Werchan and Gomez argued that, for successful abstraction and generalisation, the forgetting of irrelevant memories plays a key role (Vlach, Ankowski, & Sandhofer, 2012). As the strengthening of memories and the prevention of forgetting is an important function of sleep-based processes, a period of sleep could possibly inhibit conceptual generalisation by strengthening irrelevant memories. Wakefulness on the other hand seems to promote the forgetting of details and hence might provide a better basis for generalisation (Werchan & Gomez, 2013). This raises the question of when sleep promotes abstraction and when it does not. Werchan and Gomez (2013) suggested that it may depend on the generalisation ability of the participant, which influences the encoding of new memories. While adults are able to inhibit irrelevant information during encoding, young children cannot. Sleep-related processes may contribute, therefore, to the preservation of irrelevant details, which could slow up the abstraction of common patterns (Werchan & Gomez, 2013). Given that this notion is based on differences in the encoding process rather than the age of the participant, this hypothesis could also be applied to our results. Our category learning task presented a highly abstract and novel category to participants. In this particular situation, adult participants are in fact more like children with yet-to-be-learned familiar stimuli since, in both cases, there is limited availability of prior knowledge to distinguish between relevant and irrelevant information, which in turn might have influenced the sleep-dependent process. However, even if this novelty aspect makes an important contribution, it is unlikely to be the only factor that determines if memories show a sleep- or daytime-related enhancement, since other studies also using novel category or probabilistic structures showed sleep-dependent consolidation benefits (Djonlagic et al., 2009; Durrant et al., 2011). Another important aspect could be explicit awareness as suggested by Robertson et al. (2004), who showed that offline learning was sleep dependent for explicit skills, but time dependent for implicit skills. The importance of awareness for consolidation was also demonstrated by Song et al. (2007) by using a probabilistic variant of the serial reaction time task. This study showed that when learning occurred implicitly, sleep did not enhance general skill or sequence-specific learning. Daytime enhancement, however, occurred for general skill. Our results are in line with this hypothesis as our task was largely implicit and information-integration category learning is assumed to be mediated by an implicit, procedural system (Ashby & Maddox 2005; Smith et al., 2012). We did not observe any significant improvements for the explicit memory tasks. Differences in awareness could also explain the different results found by Djonlagic et al. (2009), since in contrast to our study, participants showed explicit knowledge of the probabilistic structure. Generally, and more importantly, these different results highlight the many open questions about the function of sleep and wake for memory consolidation.

In summary, our data show that the basic computational mechanisms in the formation of cross-modal conceptual representations are facilitated by offline consolidation across wake. Our study therefore contributes to a better understanding of the mechanisms for how representations of real-world concepts evolve over time.

## Figures and Tables

**Table 1:** Parameters of bivariate normal distributions used for the stimulus generation.

Parameter	Category 1	Category 2
*μ*_*x*_	−0.8	0.8
*μ*_*y*_	0.8	−0.8
*σ*_*x*_	2	2
*σ*_*y*_	2	2
*Ρ*_*xy*_	1.92	1.92

**Table 2:** Results of the explicit memory tasks of Experiments A and B.

	15 min Group		24 h Group		12 h day Group		12 h night Group	
	S1	S2	S1	S2	S1	S2	S1	S2
Recognition task	1.05±0.4	1.16±0.6	0.71±0.5	1.01±0.4	1.11±0.5	1.16±0.5	0.92±0.5	0.90±0.5
Association task	–	–	–	–	0.11±0.5	0.35±0.4	0.23±0.4	0.34±0.4
Categorisation task	26.9±5.1	27.2±4.6	25.9±4.1	29.3±6.2	27.4±6.6	29.54±5.7	25.4±6.0	26.70±4.6

Data for recognition and association tasks are d’±SD. Data for the categorisation task are means±SD. Correct trials for a session are out of a total of 48.

## References

[bib1] Albouy G., Sterpenich V., Balteau E., Vandewalle G., Desseilles M., Dang-Vu T. (2008). Both the hippocampus and striatum are involved in consolidation of motor sequence memory. Neuron.

[bib2] Ashby F.G., Gott R.E. (1988). Decision rules in the perception and categorization of multidimensional stimuli. *Journal of Experimental Psychology Learning Memory* and Cognition.

[bib3] Ashby F.G., Maddox W.T. (2005). Human category learning. The Annual Review of Psychology.

[bib4] Brashers-Krug T., Shadmehr E., Bizzi E. (1996). Consolidation in human motor memory. Nature.

[bib5] Dayan E., Cohen L.G. (2011). Neuroplasticity subserving motor skill learning. Neuron.

[bib6] Djonlagic I., Rosenfeld A., Shohamy D., Myers C., Gluck M., Stickgold R. (2009). Sleep enhances category learning. Learning and Memory.

[bib7] Debas K., Carriera J., Orbana P., Barakata M., Lungua O., Vanderwallea G. (2010). Brain plasticity related to the consolidation of motor sequence learning and motor adaption. Proceedings of the National Academy of Sciences of the United States of America.

[bib8] Durrant S.J., Taylor C., Cairney S.A., Lewis P.A. (2011). Sleep-dependent consolidation of statistical learning. Neuropsychologia.

[bib9] Durrant S.J., Cairney S.A., Lewis P.A. (2012). Overnight consolidation aids the transfer of statistical knowledge from the medial temporal lobe to the striatum. Cerebral Cortex.

[bib10] Ellenbogen J., Hu P., Payne J.D., Titone D., Walker M.P. (2007). Human relational memory requires time and sleep. Proceedings of the National Academy of Sciences of the United States of America.

[bib11] Fischer S., Drosopoulos S., Tsen J., Born J. (2006). Implicit learning - explicit knowing: a role for sleep in memory system interaction. Journal of Cognitive Neuroscience.

[bib12] Frankland P.W., Bontempi B. (2005). The organization of recent and remote memories. Nature Reviews Neuroscience.

[bib13] Gais S., Plihal W., Wagner U., Born J. (2000). Early sleep triggers memory for early visual discrimination skills. Nature Neuroscience.

[bib14] Gerstner J.R., Yin J.C. (2010). Circadian rhythms and memory formation. Nature Reviews Neuroscience.

[bib15] Gomez R.L., Bootzin R.R., Nadel L. (2006). Naps promote abstraction in language-learning infants. Psychological Science.

[bib16] Helie S., Waldschmidt J.G., Ashby F.G. (2010). Automaticity in rule-based and information-integration categorization. Attention Perception & Psychophysics.

[bib17] Huber R., Ghilardi M.F., Massimini M., Tononi G. (2004). Local sleep and learning. Nature.

[bib18] Jiang X., Bradley E., Rini R.A., Zeffiro T., VanMeter J., Riesenhuber M. (2007). Categorization training results in shape-and category-selective human neural plasticity. Neuron.

[bib19] Karni A., Tanne D., Rubenstein B.S., Askenasy J.J., Sagi D. (1994). Dependence on REM sleep of overnight improvement of a perceptual skill. Science.

[bib20] Krakauer J.W., Ghez C., Ghilardi M.F. (2005). Adaption to visuomotor transfomations: consolidation, inference, and forgetting. Journal of Neuroscience.

[bib21] Kumaran D., Summerfield J.J., Hassabis D., Maguire E.A. (2009). Tracking the emergence of conceptual knowledge during human decision making. Neuron.

[bib22] Kuriyama K., Stickgold R., Walker M.P. (2004). Sleep-dependent learning and motor-skill complexity. *Learning* and *Memory*.

[bib23] Lambon Ralph M.A., Sage K., Jones R.W., Mayberry E.J. (2010). Coherent concepts are computed in the anterior temporal lobes. *Proceedings of the National Academy of Sciences of the United States of America*.

[bib24] Lambon Ralph M.A. (2014). Neurocognitive insights on conceptual knowledge and its breakdown. Philosophical Transactions of the Royal Society B.

[bib25] Lau H., Alger S.E., Fishbein W. (2011). Relational memory: A daytime nap facilitates the abstraction of general concepts. PLoS One.

[bib26] Maddox W.T., Glass B.D., Wolosin S.M., Savarie Z.R., Bowen C., Matthews M.D. (2009). The effects of sleep deprivation on information-integration categorization performance. Sleep.

[bib27] McClelland J.L., McNaughton B.L., O’Reilly R.C. (1995). Why there are complementary learning systems in the hippocampus and neocortex: insights from the successes and failures of connectionist models of learning and memory. Psychological Review.

[bib28] Rogers T.T., McClelland J.L. (2004). Semantic cognition: a parallel distributed processing approach.

[bib29] Medin D., Rips L., Holyoak K.J., Morrison R.G. (2005). Concepts and categories: memory, meaning, and metaphysics. The Cambridge handbook of thinking and reasoning.

[bib30] Press D.Z., Casement M.D., Pascual-Leone A., Robertson E.M. (2005). The time course of off-line motor sequence learning. Cognitive Brain Research.

[bib31] Nemeth D., Janacsek K., Londe Z., Ullman M.T., Howard D.V., Howard J.H. (2010). Sleep has no critical role in implicit motor sequence learning in young and old adults. Experimental Brain Research.

[bib32] Reis J., Fischer J.P., Prichard G., Weiller C., Cohen L.G., Fritsch B. (2013). Time- but not sleep-dependent consolidation of tDCS-enhanced visuomotor skills. Cerebral Cortex.

[bib33] Robertson E.M., Pascual-Leone A., Press D.Z. (2004). Awareness modifies the skill-learning benefits of sleep. Current Biology.

[bib34] Robertson E.M., Press D.Z., Pascual-Leone A. (2005). Off-line learning and the primary motor cortex. Journal of Neuroscience.

[bib35] Siegel J.M. (2001). The REM sleep-memory consolidation hypothesis. Science.

[bib36] Smith J.D., Minda J.P. (2002). Distinguishing prototype-based and exemplar-based processes in category learning. Journal of Experimental Psychology Learning Memory and Cognition.

[bib37] Smith J.D., Berg M.E., Cook R.G., Murphy M.S., Crossley M.J., Boomer J. (2012). Implicit and explicit categorization: a tale of four species. Neuroscience & Biobehavioral Reviews.

[bib38] Song S., Howard J.H., Howard D.V. (2007). Sleep does not benefit probabilistic motor sequence learning. Journal of Neuroscience.

[bib39] Sweegers C.C., Takashima A., Fernandez G., Talamini L.M. (2013). Neural mechanisms supporting the extraction of general knowledge across episodic memories. Neuroimage.

[bib40] Tamminen J., Payne J.D., Stickgold R., Wamsley E.J., Gaskell G. (2010). Sleep spindle activity is associated with the integration of new memories and existing knowledge. Journal of Neuroscience.

[bib41] van der Linden M., Murre J.M. J., van Turennout M. (2008). Birds of a feather flock together: experience-driven formation of visual object categories in human ventral temporal cortex. PLoS One.

[bib42] van der Linden M., van Turennout M., Indefrey P. (2010). Formation of category representations in superior temporal sulcus. Journal of Cognitive Neuroscience.

[bib43] van der Linden M., van Turennout M., Fernandez G. (2011). Category training induces cross-modal object representations in the adult human brain. Journal of Cognitive Neuroscience.

[bib44] Vlach H.A., Ankowski A.A., Sandhofer C.M. (2012). At the same time or apart in time? The role of presentation timing and retrieval dynamics in generalisation. *Journal of Experimental* Psychology *Human Learning and Memory*.

[bib45] Wagner U., Gais S., Haider H., Verleger R., Born J. (2004). Sleep inspires insight. Nature.

[bib46] Walker M.P., Brakefield T., Morgan A., Hobson J.A., Stickgold R. (2002). Practice with sleep makes perfect: sleep-dependent motor skill learning. Neuron.

[bib47] Walker M.P., Brakefield T., Seidman J., Morgan A., Hobson J.A., Stickgold R. (2003). Sleep and the time course of motor skill learning. Learning and Memory.

[bib48] Walker M.P., Stickgold R. (2010). Overnight alchemy: sleep-dependent memory evolution. Nature Reviews Neuroscience.

[bib49] Werchan D.M., Gomez R.L. (2013). Wakefulness (not sleep) promotes generalisation of word learning in 2.5-year-old children. Child Development.

[bib50] Winocur G., Moscovitch M., Bontempi B. (2010). Memory formation and long-term retention in humans and animals: convergence towards a transformation account of hippocampal–neocortical interactions. Neuropsychologia.

